# Beyond one-size-fits-all: personalized selection of contemporary implantable cardioverter-defibrillator therapies

**DOI:** 10.1093/europace/euag200

**Published:** 2026-08-18

**Authors:** Matteo Ziacchi, Giuseppe Boriani, Mauro Biffi

**Affiliations:** Institute of Cardiology, Cardio-Thoracic and Vascular Department, IRCCS Azienda Ospedaliero Universitaria di Bologna, Via Massarenti 9, Bologna 40138, Italy; Cardiology Division, Department of Biomedical, Metabolic and Neural Sciences, University of Modena and Reggio Emilia, Policlinico di Modena, Modena, Italy; Institute of Cardiology, Cardio-Thoracic and Vascular Department, IRCCS Azienda Ospedaliero Universitaria di Bologna, Via Massarenti 9, Bologna 40138, Italy

**Keywords:** Transvenous cardioverter defibrillator, Subcutaneous ICD, Extravascular ICD, Sudden death prevention

## Abstract

Since the first human implantation in 1980, implantable cardioverter-defibrillators (ICDs) have transformed the prevention of sudden cardiac death. Today, multiple ICD platforms are available, each with distinct strengths and limitations, making individualized device selection essential to optimize outcomes and minimize complications. The transvenous ICD remains the most established technology, offering reliable defibrillation, antitachycardia pacing (ATP), and pacing support, but is limited by lead-related complications and infection risk. The subcutaneous ICD avoids intravascular leads and is particularly attractive in younger patients and those at high risk of infection, although it lacks ATP and has shorter device longevity. The extravascular ICD is an emerging alternative designed to combine the advantages of both systems by providing ATP and shock therapy without intravascular leads, though implantation is more complex and long-term evidence remains limited. Given these differences, ICD selection should be guided by patient-specific clinical characteristics rather than a one-size-fits-all approach. Key determinants include pacing indication, prior lead failure, infection risk, body mass index, age, and arrhythmic substrate. The proposed clinical pathway offers a practical framework to support tailored ICD selection by integrating these variables into a structured decision-making process. Such an individualized approach may improve patient outcomes, reduce device-related complications, and enhance the appropriate adoption of contemporary ICD technologies.

## Graphical Abstract

**Graphical Abstract:**
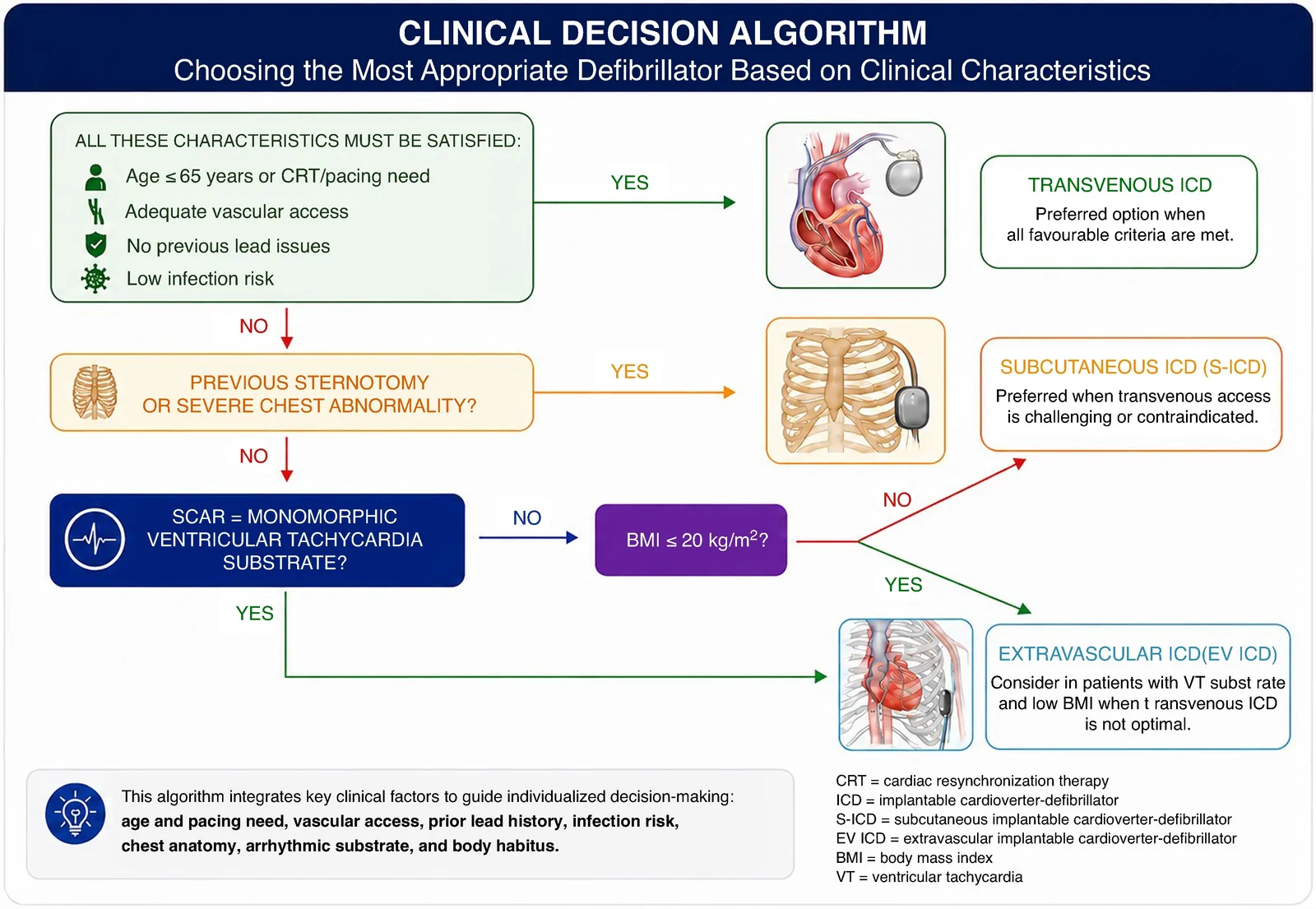

In 1970, Michel Mirowski published the first paper on the implantable cardioverter-defibrillator (ICD), and since 1980—the dawn of ICD therapy in humans—millions of lives have been saved.^1^ From early open-chest defibrillators in the 1980s, transvenous ICDs (TV-ICDs) became the cornerstone of sudden cardiac death prevention in the 1990s for over two decades, continuing to evolve from single-chamber devices to cardiac resynchronization therapy with defibrillator (CRTDs). This technology remains extremely safe and effective and progresses in refinements being the optimal choice for a substantial proportion of patients.^1^

The main strengths of TV-ICD technology are its exceptional reliability—with efficacy rates approaching 100% for terminating ventricular arrhythmias—and its ability to deliver multiple therapies: high-energy shocks for ventricular fibrillation and antitachycardia pacing (ATP) for slower re-entrant ventricular arrhythmias. In addition, TV-ICD implantation can be performed in most hospitals under local anaesthesia with relatively straightforward logistics. Finally, its comparatively low cost makes it particularly attractive from a sustainability perspective.^1^

Despite these advantages, TV-ICDs also have important limitations. Over the past two decades, it has become clear that the transvenous shocking lead is the weakest component of the system. It is prone to malfunction, especially when not optimally implanted, and lead extraction exposes patients to procedures associated with a significant risk of complications. In addition, infection remains a major concern, and its management is often complex and costly. Finally, although this applies to only a small minority of patients, implantation may be limited by lack of vascular access.^2^

In 2010, the subcutaneous ICD (S-ICD) emerged as one of the most important technological innovations in the field.^3^ Its goal was to preserve the efficacy of ICD therapy while improving safety and simplifying the management of complications. The underlying concept was to relocate the lead from the venous system to the subcutaneous space. Dedicated electrocardiographic screening should be performed before implantation to assess patient eligibility.^4^ Because arrhythmia discrimination in the S-ICD is based on analysis of a surface electrocardiogram (ECG), careful evaluation of sensing and diagnostic performance is essential. Fortunately, fewer than 10% of patients are not suitable candidates for this technology, QRS voltage and its relationship with T wave amplitude being the hardest challenge.^4^ Awareness is needed that ECG changes over time may affect long-term S-ICD eligibility, particularly in progressive cardiomyopathies where increasing scar burden reduces QRS amplitude. Although this is commonly observed in clinical practice, no robust data are currently available, owing to limited patients’ follow-up in clinical studies.

Over the past 20 years, several studies have demonstrated the non-inferiority of the S-ICD compared with the TV-ICD, establishing it as a preferred option in young patients and in those at high risk of infection—precisely the groups most vulnerable to device-related complications, although unanticipated lead failure has emerged also for the S-ICD together with unexpected battery longevity.^5^ So far, no study has shown the S-ICD to be superior to the TV-ICD, despite the improvement in implantation techniques which possibly benefits patients’ outcome.^6,7^ Management of complications has also proven simpler and safer than with transvenous systems, even though the overall complication burden has not declined substantially.^6^

The anaesthetic approach has also evolved considerably. What initially required routine anaesthesiologist support and endotracheal intubation can now often be managed with deep sedation combined with locoregional nerve blocks, which may also be performed by non-anaesthesiologist operators.^8^

Two important limitations remain. The first is device longevity, which, although improved in newer generations, remains inferior to that of TV-ICDs. The second is the lack of ATP capability. Both limitations are currently being addressed through the development of modular systems, improved battery performance, and alternative circuit designs. Device cost also remains higher than that of the TV-ICD.

Eventually, an S-ICD recipient with recurrent monomorphic VT may be preferentially treated by VT ablation and/or antiarrhythmic drugs rather than by addition of a leadless unit to deliver ATP.

The number of S-ICD implantations has increased substantially over time, although it likely remains lower than the number of patients who could potentially benefit from this technology.

More recently, a new platform has entered clinical practice: the extravascular ICD (EV-ICD). The aim of this novel technology is to combine the advantages of both TV- and S-ICD systems. In other words, it seeks to provide enhanced device longevity compared to the S-ICD with the same size of a transvenous device, the ability to deliver both ATP and shocks, and the capacity to prevent asystole.^9^ These features are achieved without intravascular leads, thereby simplifying and potentially reducing the risks associated with infection and lead-related complications.

To achieve this, the lead is placed in the anterior mediastinum beyond the sternum, making the procedure currently more complex than either TV- or S-ICD implantation. Patient eligibility is not universal: at present, the procedure is contraindicated in patients with prior sternotomy or a history of pericardial disease. In addition, pre-procedural chest computed tomography (CT) imaging is recommended to assess patient-specific anatomy and plan reference makers for the lead pathway.^9^

The EV-ICD implantation generally requires general anaesthesia, although early experience with deep sedation—similar to that used for S-ICD implantation—is emerging. The presence of a cardiac or thoracic surgeon on site was recommended in the early experience, but it becomes unnecessary once familiarity with the procedure has been acquired under expert proctorship. Indeed, an excellent safety profile is reported, without long-term sequelae in either the registration study or the observational registry, efficacy of shock and ATP therapy being comparable to that of other ICD systems.^9,10^

Nevertheless, it is undeniable that both pre-implant evaluation and implantation logistics remain more complex than with alternative technologies, and no direct comparative studies with other ICD platforms are yet available. The EV-ICD is a technology with huge potential, likely more comparable to the TV-ICD than to the S-ICD, but additional data are still needed to better define its clinical role. The ATP efficacy, the size, and its longevity make the EV-ICD particularly attractive for paediatric as well as for patients with low body mass index (BMI).^11^ One of the limitations to the widespread adoption of this technology may be the rate of inappropriate shocks, which, in the pivotal trial, was higher than that reported with other defibrillation technologies. On the other hand, new technologies often initially carry a greater risk of adverse events compared with established standards of care; however, this risk tends to decrease over time as a result of software improvements and the accumulation of operator experience through an appropriate learning curve. This phenomenon has also been observed in the past with both TV-ICDs and S-ICDs, whose early generations exhibited rates of inappropriate shocks comparable to those currently reported for the EV-ICD. Consistent with this trend, outcomes have improved over time for the EV-ICD as well, as evidenced by the lower rate of inappropriate shocks observed during the latter phase of the pivotal trial compared with the earlier phase.^9–11^

More recently, the ASCEND EV study evaluated a novel extravascular parasternal ICD. This system uses an extravascular lead positioned along the left parasternal border to deliver defibrillation and pacing therapy from outside the heart. The system has been shown to be feasible and safe; however, further evidence is needed before it can be widely adopted in routine clinical practice.^12^ Additional data are expected from the ALARION-EV study (NCT07324031), which will likely provide the evidence required to support its broader clinical use.

The flow chart reported in *Figure 1* is proposed as a practical tool to assist physicians in the selection of the most appropriate type of ICD based on patient’s individual characteristics (*Figure 1A*).

**Figure 1:**
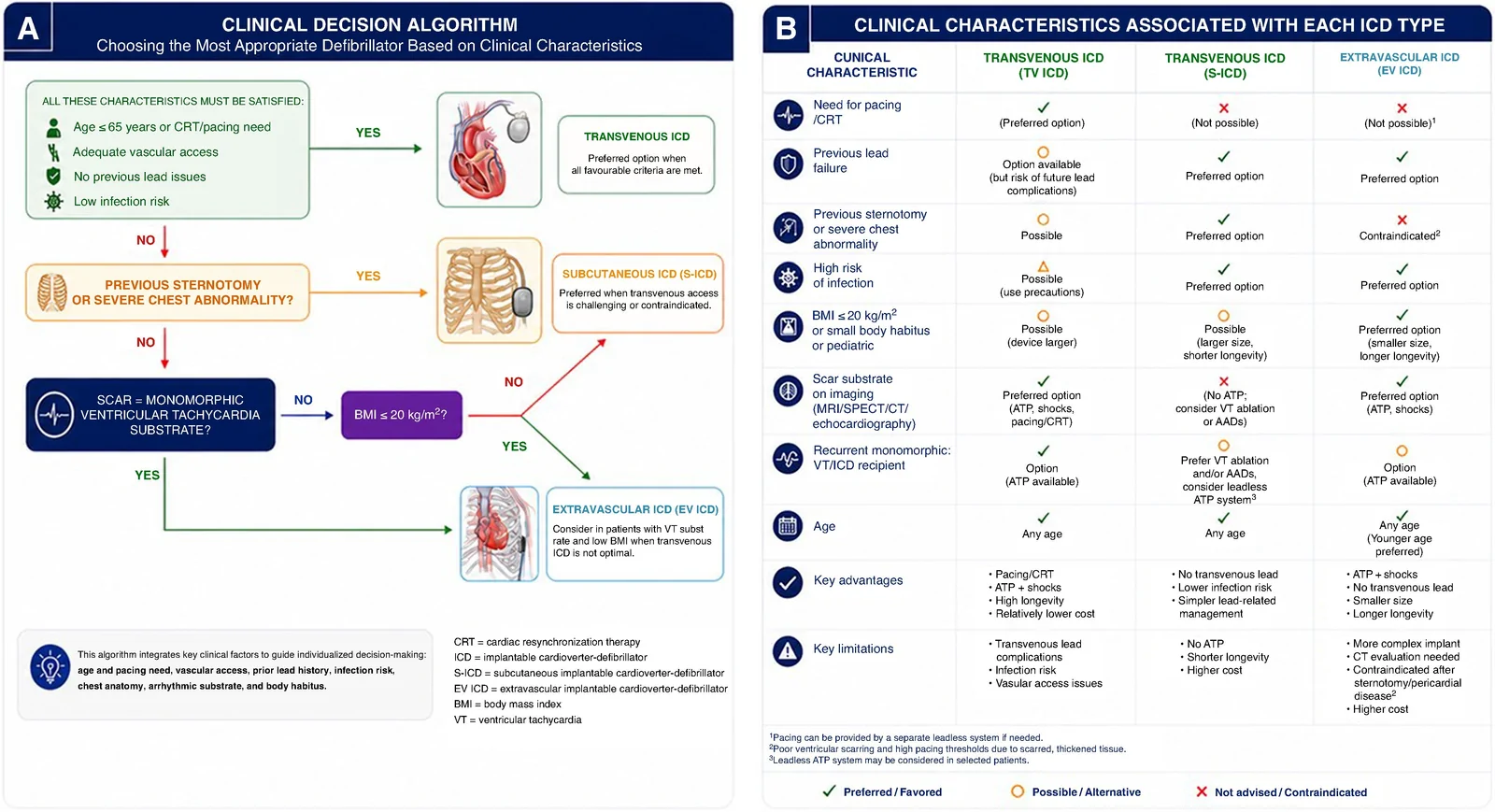
Integrated overview of patient selection for ICD therapy. (*A*) is clinical decision algorithm for selecting the most appropriate ICD based on key patient characteristics; (*B*) is a comparative summary of clinical characteristics associated with each ICD type, including advantages, limitations, and clinical considerations.

When cardiac resynchronization therapy (CRT) or conventional atrial and/or ventricular pacing is required, transvenous systems remain the preferred option, although combinations of non-communicating devices are increasingly reported. Conversely, in patients without superior venous access, pairing a non-TV-ICD with leadless pacing or CRT offers an attractive alternative, particularly after prior cardiac surgery.^13^ Previous sternotomy is generally a contraindication to EV-ICD implantation because it increases procedural complexity and, more importantly, is associated with poor ventricular sensing and high pacing thresholds due to scarred, thickened tissue over the right ventricular free wall. Overall, only a minority of patients meeting ICD criteria also require pacing for conduction disease; first-degree atrioventricular block and prior cardiac surgery are the strongest predictors.^14^ In patients without pacing or CRT indications, previous transvenous lead failure—or a high anticipated risk of lead complications related to chest anatomy or physical activity—favours selection of a non-transvenous system.

Assessing infection risk, though not straightforward, is also critically important: previous cardiac implantable electronic device CIED infection or lead/valve endocarditis, dialysis, or end-stage kidney disease clearly favours a non-TV-ICD.^15^

Young age and small body habitus favour the EV-ICD owing to its smaller size and longer projected longevity compared with the S-ICD.

The underlying cardiac substrate also plays an important role in ICD selection: the presence of myocardial scar at MRI/SPECT/CT/echocardiography represents the substrate for re-entrant monomorphic ventricular tachycardia amenable to termination by ATP^16^ independently of disease aetiology. In these patients, EV-ICD and TV-ICD are likely the preferred options, with a preference for non-transvenous technology in younger individuals. Though predicting ATP efficacy remains challenging, evidence supporting its high termination rate and low risk of acceleration with multiple attempts makes it an essential component of ICD therapy in patients with scar-related myocardial substrate, whose clinical arrhythmia is monomorphic VT in >90% of cases.^17^ Antitachycardia pacing capability should hence be available from the outset of patients’ treatment in this clinical setting.

Conversely, in the absence of myocardial scar, a subcutaneous technology can be favoured based on patient physical habit and age at presentation.

*Figure 1B* summarizes clinical characteristics associated with each ICD type.

In conclusion, several ICD technologies are now available, all with excellent safety and efficacy profiles, but each with distinct strengths and limitations. Current ESC/EHRA guidelines reserve non-transvenous technologies for specific subgroups of patients, thereby limiting their broader use. However, in light of the growing body of evidence supporting the efficacy and safety of the various defibrillation technologies, it may be time to move beyond considering non-transvenous systems as merely a second-line option and instead regard them as equivalent alternatives to transvenous systems in a much wider range of clinical settings. For this reason, careful selection of the most appropriate device for each individual patient remains essential.
